# High prevalence of *Sarcocystis calchasi* in racing pigeon flocks in Germany

**DOI:** 10.1371/journal.pone.0215241

**Published:** 2019-04-15

**Authors:** Sylvia L. Parmentier, Kristina Maier-Sam, Klaus Failing, Achim D. Gruber, Michael Lierz

**Affiliations:** 1 Clinic for Birds, Reptiles, Amphibians and Fish, Justus Liebig University Giessen, Giessen, Germany; 2 Unit for biomathematics and data processing, Justus Liebig University Giessen, Giessen, Germany; 3 Department of Veterinary Pathology, Freie Universität Berlin, Berlin, Germany; Charles University, CZECH REPUBLIC

## Abstract

The apicomplexan parasite *Sarcocystis calchasi* (Coccidia: Eimeriorina: Sarcocystidae) is the causative agent of Pigeon Protozoal Encephalitis (PPE) and infects birds of the orders Columbiformes, Piciformes and Psittaciformes. *Accipiter* hawks (Aves: Accipitriformes) are the definitive hosts of this parasite. Infections of *S*. *calchasi* have been detected in Germany, the United States and Japan. However, the prevalence of the parasite in racing pigeon flocks has not yet been determined. Here, the first cross-sectional prevalence study to investigate *S*. *calchasi* in pigeon racing flocks was accomplished including 245 pigeon flocks across Germany. A total of 1,225 muscle biopsies, were taken between 2012 and 2016 and examined by semi-nested PCR for *S*. *calchasi* DNA targeting the ITS gene. Additionally, a questionnaire on construction of the aviary as well as management and health status of the flock was conducted. In 27.8% (95% C.I. = 22.3–33.8%) of the flocks, *S*. *calchasi* DNA was detected in at least one pigeon. Positive flocks were located in 15 out of 16 federal states. A significant increase of infected racing pigeons was seen in spring. Half-covered or open aviary constructions showed a trend of increase of the prevalence rate, while anti-coccidian treatment and acidified drinking water had no effects. The high prevalence and the geographical distribution of *S*. *calchasi* suggest a long-standing occurrence of the parasite in the German racing pigeon population. For pigeons presented with neurological signs or other symptoms possibly related to PPE, *S*. *calchasi* should be considered as a potential cause throughout Germany.

## Introduction

In 2006 several racing pigeons (*Columba livia* f. *domestica*) (Aves: Columbiformes) from different racing pigeon flocks in the area of Berlin, Germany, developed clinical signs of diarrhoea, polyuria, opisthotonus, torticollis, and paralysis [1]. Histopathology revealed varying degrees of multifocal to coalescing, granulomatous and necrotizing encephalitis and myositis with sarcosporidian cysts [1]. A new *Sarcocystis* species, *Sarcocystis calchasi* (*S*. *calchasi*) (Coccidia: Eimeriorina: Sarcocystidae) was identified and described as the causative agent of Pigeon Protozoal Encephalitis (PPE) [2]. Like all *Sarcocystis* species [3,4], *S*. *calchasi* has a heteroxenous two-host life cycle [2]. In addition to the racing pigeon, the intermediate host spectrum includes the Common Wood Pigeon (*Columba palumbus*) [5], the White-winged Dove (*Zenaida asiatica*), the Eurasian Collared Dove (*Streptopelia decaoto*) [6], and various psittacine species [7,8]. DNA of *S*. *calchasi* was also detected in the European Green Woodpecker (*Picus viridis*), and the Great Spotted Woodpecker (*Dendrocopos major*) [9]. *Accipiter* hawks (Aves: Accipitriformes) were confirmed as the definitive hosts of *S*. *calchasi* [10,11]. Infections of *S*. *calchasi* have been recorded in Germany, the United States of America, and Japan [2,12–14], suggesting a worldwide distribution of the parasite.

*Sarcocystis calchasi* causes a dose-dependent biphasic disease in pigeons [10,15]. It was demonstrated in experimentally infected pigeons, high infectious doses (>10^4^ sporocysts) are lethal between 7- and 12-days post infection (dpi) during the first acute state of the disease. Mid-level infected birds (10^3^ to 10^4^ sporocysts) develop apathy and polyuria in this phase, while low-level infected birds (10^2^ sporocysts) do not show any clinical signs at this time [15]. The acute phase, if survived, is followed by an asymptomatic period until neurological signs appear around 51 dpi [15]. Pigeons infected with low parasite numbers may only show neurological signs during the chronic phase [15]. The clinical signs resemble those of an infection with *Salmonella* Typhimurium var. Copenhagen (*S*TVC), or avian Paramyxovirus-1 (APMV-1) which commonly cause neurologic disease in pigeons [16,17]. Experimental infection of cockatiels (*Nymphicus hollandicus*) with *S*. *calchasi* also resulted in a biphasic disease with central nervous signs which mirrored PPE in many aspects but with a more diverse pathology and no dose-dependency [7].

The life cycle of *S*. *calchasi* is completed with *Accipiter* hawks as the definitive hosts [2]. The Northern goshawk (*Accipiter gentilis gentilis*; hereafter goshawk), and the Eurasian sparrowhawk (*Accipiter nisus nisus*; hereafter sparrowhawk) widely occur across Germany [18,19] and are regarded as synanthrophic birds [18,20]. Both the goshawk and the sparrowhawk prey on pigeons and are frequently observed in close proximity of pigeon flocks [21–26]. Racing pigeons may represent up to 30% of their prey [27]. *Accipiter* hawks commonly sit on top or near by the lofts waiting for their prey. They are considered to introduce *S*. *calchasi* into the flock via defecation into the aviaries. Thus, a high risk of a *S*. *calchasi* infection in the pigeons can be anticipated [11]. Reduced performance in races or breeding may be the consequence. This cross-sectional study was designed to determine the prevalence of *S*. *calchasi* in the racing pigeon population throughout Germany. Based on the current prevalence, a risk assessment for German racing pigeon flocks can be conducted, and the common awareness of the disease may be enhanced. Adaption of loft construction design and other preventative measures are recommended and the importance of PPE as differential diagnosis in pigeons with neurologic signs is underlined.

## Material and methods

### Study design

#### Background

Racing pigeons are domesticated animals, without a natural but rather a man-made distribution. They are kept in dense flocks, increasing the risk that many animals may get infected with *S*. *calchasi* via contaminated faeces from *Accipiter* hawks. While racing pigeons are usually kept in enclosed compartments, breeding pairs are commonly kept in semi-open aviaries [28]. Since the risk of faecal contamination by *Accipiter* hawks is expected to be higher in those aviaries, breeding pigeons were selected for the present study [29]. In the aviaries many birds may get in contact with contaminated faeces defecated by *Accipiter* hawks into the aviary. Hence, high prevalence within one flock above 50% is assumed. Therefore, a cross-sectional study to estimate a flock-based prevalence is preferable to an individually-levelled prevalence.

#### Calculation of sample size

The required sample size per flock was calculated by applying the tables of Cannon and Roe, based on the assumption that the flock contains typically 50 pigeons [30]. Demanding a 0.95 probability to find at least one positive case in the flock sample and assuming a prevalence of 50% within the flock (resulting inequality P(X ≥ 1 | Prevalence within flock = 0.50) ≥ 0.95 where X = number of positive pigeons in the sample) the appropriate sample size per flock was calculated as n = 5 [30].

To estimate the number of flocks required for the cross-sectional study two conditions were determined: First, the 95% confidence interval (95% C.I.) of the estimated flock prevalence should have an accuracy of ± 5%, and second, the flock prevalence was expected to be approximately 20% (= design prevalence). According to the tables of Cannon and Roe, these premises resulted in a total sample amount n = 245 flocks [30].

#### Selection of participants

The distribution of the sampled flocks depended on the inclusion of all federal states of Germany, and the density of racing pigeon flocks in the particular region. Therefore, a two-step stratification approach was used to select the flocks. The first stratification was based on all non-city states (n = 13); city-states (Berlin, Bremen, Hamburg) were included in the surrounding state. Assuming again a flock prevalence of 20% the probability of detecting *S*. *calchasi* at least in one sampled flock per federal state was defined to be at least 90% (resulting inequality: P(X ≥ 1 | Prevalence = 0.20) ≥ 0.90) which resulted (again according to the tables of Cannon and Roe) in a minimum sample size of n = 11 flocks per federal state [30]. Under this premise, 143 (13 federal states x 11 flocks) samples were initially set (Table 1, Column 5). The remaining 102 flocks required for reaching the total amount of 245 flocks were selected by a second stratification which was based on the number of racing clubs in each federal state. For this purpose, a register of members, contributed by the Federation of German Racing Pigeon Breeders, was arranged by place of residence (federal state-based) and subsequently by racing clubs. The 102 flocks were distributed in proportion to the number of racing clubs in the respective state (Table 1, Column 6). The racing clubs to be sampled were selected randomly using the random number function of the program Excel. By relative comparison of the sample size of n = 245 with the total amount of racing pigeon associations in Germany (n = 683) the average selection rate was 0.36 flocks per racing club. Within the selected racing clubs, breeders were also randomly selected again by Excel. If a selected breeder did not agree with the investigation or did not house his breeding pigeons in an aviary, an alternative breeder was randomly selected. The breeders arbitrarily selected 5 birds out of all birds present in their aviary from all birds present in the aviary for at least 3 months.

**Table 1:** Sampling design for the determination of flock prevalence in racing pigeon.

Federal State	Flocks	Racing Clubs (RC)	Share in % of total number in Germany	Minimum Sample Size per Federal State	Surcharge based on RC	Total Sample Size
Baden-Wuerttemberg	1,489	47	6.9	11	7	18
Bavaria	3,139	91	13.3	11	14	25
Brandenburg / Berlin	967	18	2.6	11	3	14
Hesse	2,524	65	9.5	11	10	21
Mecklenburg-Western Pomerania	853	20	2.9	11	3	14
Lower Saxony / Bremen	4,223	110	16.1	11	16	27
North Rhine-Westphalia	10,294	217	31.8	11	33	44
Rhineland-Palatine	624	20	2.9	11	3	14
Saarland	377	10	1.5	11	1	12
Saxony	589	14	2.1	11	2	13
Saxony-Anhalt	1,274	23	3.4	11	3	14
Schleswig-Holstein / Hamburg	1,064	26	3.8	11	4	15
Thuringia	1,123	22	3.2	11	3	14
Total	28,540	683	100.0	143	102	245

### Statistics

A flock was counted positive if DNA of *S*. *calchasi* was detected in at least one sample. Confidence intervals of the total flock prevalence as well as those for the different federal states were calculated. As information regarding factors that may influence the prevalence the following items were collected via a questionnaire: the construction of the aviaries (roof and side panel), standard treatment against *Eimeria* spp. (e.g. toltrazuril, sulphonamide, oregano oil, chamomile flower extract), acidification of the drinking water, faecal abnormalities, breeding performance reduction, Young Pigeon Disease Syndrome (YPDS), unusually high losses of pigeons in a race, breeding pigeons with neurological symptoms, outbreaks of *S*TVC or APMV-1, and vaccination against AMPV-1. Additionally, geographic data was used: federal state, region, area (km^2^) of the postal code area (PCA) which was sampled, population of the PCA, density of population of the PCA, racing pigeon breeders in the PCA, racing pigeon breeders/area (km^2^) of the PCA, and share of racing pigeon breeders in the population of the PCA. Size of the flock, season and year of sampling were further variables. All in all, 23 variables were included into the statistical analysis. With regard to the high number of these variables the appropriate statistical analysis was performed in two steps. In the first step the raw statistical associations between the target variable and the independent variables are described by frequency tables and examined either by simple logistic regression (metric variables) or chi-square-test (nominal variables). Resulting from this in the second step a multiple logistic regression model was fitted to the data in a stepwise manner to consider possible overlapping effect of the variables. Hereby, first the most important influencing variable is incorporated in the logistic model. Then, in the following steps, further variables can be incorporated into the model if their statistical criterion for inclusion is given (step selection by maximum likelihood ratio, MLE criterion). The procedure stops if all further variables are insignificant.

The data were analysed with the statistical software package BMDP Dixon [31].

Frequency tables were generated with the program BMDP4F and 95% confidence intervals (CI) were build. Additionally, the exact Fisher and Chi-Square-Tests were performed in dependence of the minimum expected value. The simple and multiple stepwise logistic regression was performed by the program BMDPLR. In all cases, p-values of < 0.05 were assessed as statistically significant (significance level).

### Sample collection

Pigeons were sampled either in the clinic for birds, reptiles, amphibians and fish, Justus Liebig University Giessen, Germany, or locally at the breeder’s place as part of the veterinary health monitoring, which was offered to the breeders by the clinic, between 2012 and 2016. Consultation included a control of the flocks, regarding structural defects and suggestions of enhancement, check of the hygiene protocol and standards, inspection of foods and additives with evaluation of quality and need. At suspicion, parasitological control of faecal samples, investigation of ectoparasites or other skin related diseases, and control of nodular, joint associated alteration related to *S*TVC took place. For the control of an infection with *S*. *calchasi*, pigeons were placed under general anaesthesia (isoflurane, Isothesia, Henry Schein, Abbott Labarotories Ltd., Berkshire, UK), and analgesia (1 mg/kg meloxicam, Meloxivet 1.5 mg/ml, Elanco Animal Health, Basingstoke, UK). From each pigeon, a biopsy of the size of a rice kernel (approximately 5 x 2 x 2 mm) was taken from the upper breast muscle (*Musculus pectoralis superficialis*) and immediately transferred on ice packs. The site of the biopsy was sutured with one simple interrupted suture; skin was closed by a vertical mattress stitch (Coated Vicryl, 5–0, ETHICON, Johnson & Johnson, Somerville, US). All birds recovered and were brought back into their aviaries by their owners. Breeders did not report impairments of the birds afterwards.

### Semi-nested polymerase chain reaction specific for *S*. *calchasi* DNA

DNA was extracted (DNeasy Blood & Tissue Kit; Qiagen, Hilden, Germany) from the muscular biopsies according to manufacturer instructions. DNA concentration was measured (NanoDrop 2000c Spectrophotometer; Thermo Fisher Scientific, Wilmington, DE, USA) and, if necessary, diluted to < 5ng/μl. The semi-nested PCR specific for *S*. *calchasi* DNA targeting the internal transcribed spacer (ITS) gene was performed as described previously [32]. The first amplification was conducted with primers SCa1 (3’-CTCCTTGZTCGAGAATGAACATGAG-5’) and SCa2 (3’-GATCATCTTTTCGACGACAATATCG-5’). The second amplification used primers SCa1 and SNCa3 (3’-TCCAGAGAAGATCCCCTGGCTAC-5’). A sample of DNase-free water was added to each run and served as negative control. As positive control, DNA was extracted from the Berlin strain of *S*. *calchasi* and added as a serial ten-fold dilution [11]. Thus, the sensitivity of the assay was constantly monitored. For confirmation of specificity, PCR products of positive samples were regularly controlled by sequencing. DNA was purified (GeneJet PCR Purification Kit, Thermo Fisher Scientific) and sequenced by a commercial DNA sequencing service (LGC Genomics GmbH, Berlin, Germany) using the reverse primer SNCa3. Subsequently, the sequences were compared to all sequences listed in GenBank using the BLAST program (http://blast.ncbi.nlm.nih.gov/Blast.cgi).

## Results

### Sample collection and questionnaire

A total of 1,225 samples were collected from 245 flocks with 5 samples per flock in the period between 2012 and 2016. Gender distribution between the sampled animals was 574 (46.9%) female, and 651 (53.1%) male pigeons. The sample collection took place mainly from October until March (189/245 = 77.1%). The flock size varied from 10 to 300 birds (mean 94.6). On average, the lofts had been in their current locations for 36 years (range 1–135 years). A differentiation was made between different construction types of the aviaries. Fully covered (74), semi-covered (35), and uncovered (136) aviaries were distinguished. The majority of aviaries (226) had three sides closed with tight-net wire, 10 had two, and 8 aviaries had only one side constructed with wire. One aviary had no side constructed with wire but was uncovered. Further results of the questionnaire are listed in Table 2. The population density of the communities where the pigeon husbandries were located ranged from 46.4 people/km^2^ (P/km^2^) to 580.8 P/km^2^. The density of pigeon breeders varied from 0.008 breeders/km^2^ (B/km^2^) to 4.16 B/km^2^.

**Table 2:** Results of the questionnaire of 245 racing pigeon breeders in the period between 2012 and 2016.

Variable	Yes	(%)	No	(%)	No Answer	(%)
Anti-parasitic treatment with an effect on *Eimeria* spp.	109	44.5	136	55.5	0	0
Acidification of drinking water	180	73.5	65	26.5	0	0
Faecal abnormalities	53	21.6	192	78.4	0	0
Breeding performance reduction	39	15.9	206	84.1	0	0
Young Pigeon Disease Syndrome	129	52.7	111	45.3	5	2.0
Severe losses during racing flights	93	38.0	152	62.0	0	0
Neurological signs	27	11.0	218	89.0	0	0
APMV-1 Infection	2	0.8	243	99.2	0	0
*S*TVC-Infection	9	3.7	236	96.3	0	0
Vaccination against APMV-1	239	97.6	5	2.0	1	0.4

### Semi-nested polymerase chain reaction specific to *S*. *calchasi*

In total, 68/245 (27.8%; 95% C.I. = 22.3–33.8%) of the flocks were positive for *S*. *calchasi* DNA by semi-nested PCR (Table 3). In 52/68 (76.5%) positive flocks, DNA of *S*. *calchasi* was identified in one out of five pigeons. Out of 68 flocks, 12 (17.6%) had two birds which tested positive and 2 (2.9%) had three or four birds which tested positive.

**Table 3:** Prevalence of *S*. *calchasi* in different federal states.

Federal States	Positive tested flocks/ Number of sampled flocks	Percentage (%)	0.95 C.I. (%)
Baden-Württemberg	4/18	22.2	6.4–47.6
Bavaria	8/25	32.0	14.9–53.5
Brandenburg / Berlin	3/14	21.4	4.7–50.8
Hesse	1/21	4.8	0.1–23.8
Mecklenburg-Western Pomerania	7/14	50.0	23.0–77.0
Lower Saxony / Bremen	14/27	51.9	31.9–71.3
North Rhine-Westphalia	16/44	36.4	22.4–52.2
Rhineland-Palatine	2/14	14.3	1.8–42.8
Saarland	6/12	50.0	21.1–78.9
Saxony	0/13	0	20.6
Saxony-Anhalt	3/14	21.4	4.7–50.8
Schleswig-Holstein / Hamburg	3/15	20.0	4.3–48.1
Thuringia	1/14	7.1	0.2–33.9
**TOTAL**	**68/245**	**27.8**	**22.2–33.8**

### Flock specific factors correlating with the occurrence of *S*. *calchasi*

**Fig 1:**
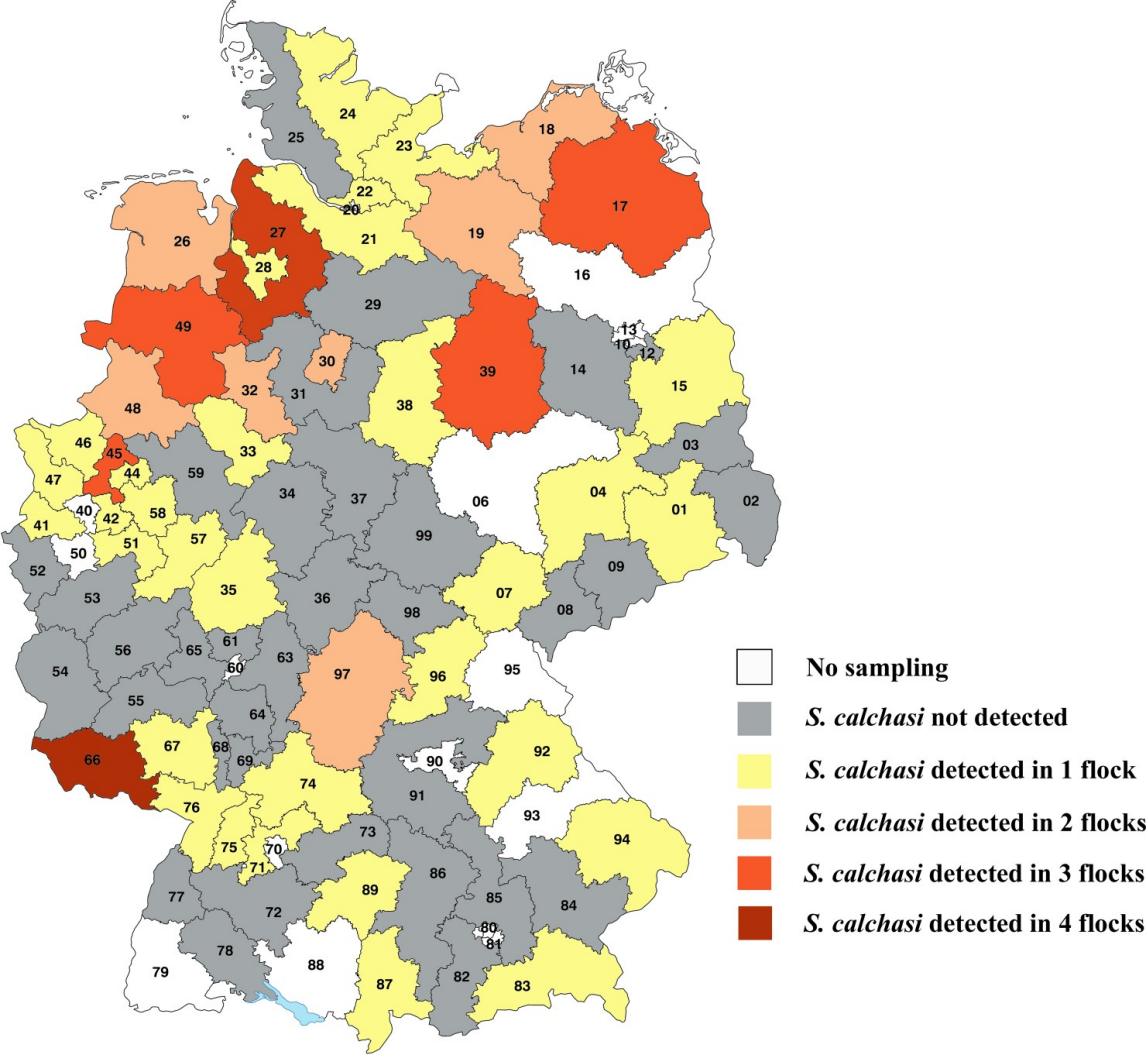
Detection of *S*. *calchasi* DNA in racing pigeon flocks by division into districts. Map of Germany fielded by postal codes.

**Fig 2:**
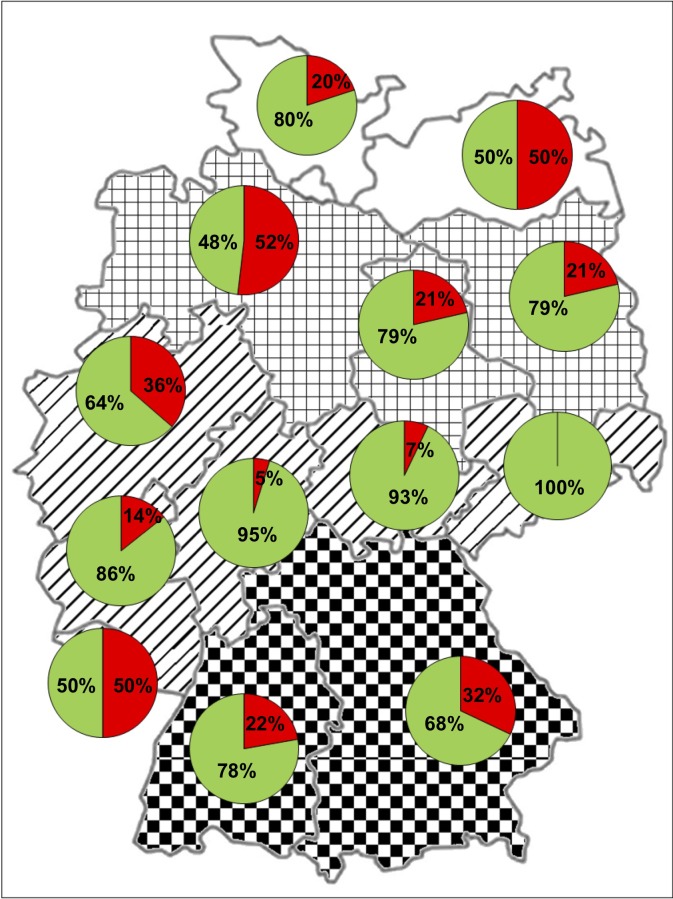
Distribution of *S*. *calchasi* DNA positive (red) and negative (green) racing pigeon flocks in percentages throughout Germany, using geographical division into regions. Mecklenburg Coastal Lowland (white) and the North German Plain (squared) had a significantly higher prevalence (p = 0.0081) than the Central Upland and Scarplands on either sides of the Rhine Valley (fasciated), and the Alpine Forland (checker).

Out of the 23 variables tested, the factors ‘federal state’, ‘region’ and ‘season’ were associated with the occurrence of *S*. *calchasi* with statistical significance (Table 4; raw associations). *Sarcocystis calchasi* was detected in at least one flock in 12 out of 13 non-city federal states (Fig 1, Table 3). Including the city states, a distribution in 15 of 16 federal states in Germany was demonstrated. The distribution of the prevalence of *S*. *calchasi* in Germany differed from a random distribution (federal state p = 0.0013) (Table 4). While Hesse (4.8%; 95% C.I. = 0.1–23.8%) and Thuringia (7.1%; 95% C.I. = 0.2–33.9%) had a low prevalence, it was high in Mecklenburg-Western Pomerania (50.0%; 95% C.I. = 23.0–77.0%), Saarland (50.0%; 95% C.I. = 21.1–78.9%), and Lower Saxony (51.9%; 95% C.I. = 31.9–71.3%). Regarding state-overlapping geographical areas the prevalence varied again significantly (p = 0.0081): flocks in the Mecklenburg Coastal Lowland and the North German Plain had a higher prevalence of *S*. *calchasi* than the Central Upland, Scarplands on either sides of the Rhine Valley, and the Alpine Forland (Fig 2).

**Table 4:** Identification of correlating variables–p-Values of the simple logistic regression or chi-square-test considering each variable separate (raw associations). Variables partially log-transformed (lg) if their distribution was skewed to the right.

Variable	P-Value
Federal state	0.0013*
Region	0.0081*
Area (km^2^) of the postal code area	0.84
lgPopulation of the postal code area	0.59
lgDensity of population of the postal code area	0.92
lgRacing pigeon breeders in the postal code area	0.39
lgRacing pigeon breeders/area (km^2^) of the postal code area	0.89
lgShare of racing pigeon breeders in the population of the postal code area	0.76
Season	0.0031*
Year	0.40
lgSize of the flock^Q^	0.47
Construction of the roof of the aviary^Q^	0.29
Construction of the side panels of the aviary^Q^	0.70
Anti-parasitic treatment with an effect on *Eimeria* spp.^Q^	0.35
Acidification of drinking water^Q^	0.34
Faecal abnormalities^Q^	0.81
Breeding performance reduction^Q^	0.22
Young Pigeon Disease Syndrome^Q^	0.90
Severe losses during racing flights^Q^	0.96
Neurological signs^Q^	0.82
APMV-1-infection^Q^	0.38
*S*TVC-infection^Q^	0.70
Vaccination against APMV-1^Q^	0.16

* Statistically significant association to the occurrence of *S*. *calchasi*; ^*Q*^ Answer determined by questionnaire

The season when flocks were sampled showed a significant influence in the rate of positive flocks (p = 0.0031) (Table 4 and Table 5). Samples collected in April, May, or June had a significantly higher percentage of positive test results.

**Table 5:** Higher detection rate of *S*. *calchasi* DNA in the second quarter of the year.

PCR result	January, February, March	April, May, June	July, August, September	October, November, December
Positive	19 (19.2%)	11* (61.1%)	11 (28.9%)	27 (30.0%)
Negative	80 (80.8%)	7 (38.9%)	27 (71.1%)	63 (70.0%)

* Statistically significant association to the occurrence of *S*. *calchasi*

Construction of the roof (p = 0.29) and side panels of the aviaries (p = 0.70) did not affect the results significantly, of the 16 flocks with multiple positive pigeons, ten flocks were housed in open or half-covered aviaries, while six flocks had a fully covered roof. Usage of anti-parasitic medication (p = 0.35) and acidified drinking water (p = 0.34) were not correlated with the detection of *S*. *calchasi* either (Table 5). Positive flocks were detected in rural areas (density of 206.0 P/km^2^) as well as in urban areas (density 3542.8 P/km^2^). The infection rate was not correlated with the breeding performance of the flocks (p = 0.22). All flocks that tested positive were located in the same area for more than three years (Table 6).

**Table 6:** Detection of *S*. *calchasi* compared with the years of local racing pigeon breeding history.

Year	*S*.* calchasi*				Total
	Negative	Percentage (%)	Positive	Percentage (%)	
1–2	4	100	0	0	4
3–5	8	66.7	4	33.3	12
6–10	9	81.8	2	18.2	11
11–20	23	67.6	11	32.4	34
21–30	26	76.5	8	23.5	34
>30	103	72.0	40	28.0	143
No Answer	4	57.1	3	42.9	7

Data were collected via questionnaire

### Multiple stepwise logistic regression

By means of the multiple stepwise logistic regression procedure as described above, no substantial changes of the associations between the target variable and the independent variables were shown. 'Region' and 'Season' retained their significant effect in combination, while the other tested factors, even when combined in the model, showed again no significant effect on the detection rate of *S*. *calchasi*. The influencing variable ‘federal state’ was excluded of the multiple stepwise logistic regression due to the small number of cases in the individual federal states.

## Discussion

In the present study an almost country-wide occurrence of *S*. *calchasi* in the racing pigeon population was demonstrated. However, the prevalence regarding the federal states differed substantially. In Saxony, where all flocks were tested negative, a low pigeon breeder density with a mean of 0.1 B/km^2^ is present. In regions with a low pigeon population, *Accipiter* hawks may not prey regularly on pigeons and use other avian and non-avian species [20–22,24,33]. The higher the density of breeders, as seen, the more likely is a steady contact over time between *Accipiter* hawks and racing pigeons which is demonstrated for example in North-Rhine Westphalia with a high density of breeders (average 0.8 B/km^2^) and a high prevalence of *S*. *calchasi* (36.4%). The notion that the prey preference of the *Accipiter* hawks is essential for the occurrence of *S*. *calchasi* in the local pigeon population is supported by the fact that all positive flocks were located in the same area for at least three years, which allowed for an adaptation of the raptor to the local feeding source. A similar conclusion was drawn by Parmentier et al. [5] regarding the population density of the common wood pigeon which was determined as natural reservoir of *S*. *calchasi*. Therefore, the occurrence of *S*. *calchasi* in racing pigeons and wood pigeons may be dependent on the prey preferences of the local *Accipiter* hawks.

Data suggest a seasonal peak in the second quarter of the year which may correlate with the hunting behaviour of *Accipiter* hawks. *Accipiter* hawks prey increasingly on columbids during winter and early spring [23,34–38], due to the absence of migratory birds. While sampling the pigeons, many breeders mentioned a periodical visit of goshawks or sparrowhawks to their aviaries, mainly in winter and spring time. Therefore, the transmission of faeces into the aviaries seems likely and the accumulation of contaminated faeces during that period would increase the risk of infection for the pigeons, particularly towards the beginning of spring. Since the pigeons that were experimentally infected with *S*. *calchasi* had been tested positive by PCR for at least two months [39], positive tested animals are supposed to accumulate during winter and early spring and numbers would be highest in the following months. An increased infection rate during early spring would explain the higher prevalence of positive pigeons in the months of April to June.

Rooftops are preferred landing sites of free ranging birds including *Accipiter* hawks. In the case of open and half-covered roofs, *Accipiter* hawks can more easily defecate into the aviary, which presumably enhances the risk of transmitting *S*. *calchasi* sporocysts. A significant increase of the flock-based prevalence in open or half-covered aviaries was not recorded but an increase of the prevalence in the separate flocks was seen: 16 flocks had more than one pigeon that tested positive for *S*. *calchasi* DNA, of these, 62.5% were sampled in open or half-covered aviaries. This may indicate a tendency towards a correlation between the design of the aviary and the prevalence of *S*. *calchasi* DNA in the pigeons. As discussed previously, sporocysts of *S*. *calchasi* might passage through the intestinal tract of a pigeon without excysting and therefore still be contagious in the pigeon faeces [15]. In our survey this might have played an additional role in the flock-based prevalence. In contrast, there was no correlation between standard anti-parasitic treatment with an effect on *Eimeria* spp. and the occurrence of *S*. *calchasi* infections, consistent with previous results [39]. *Sarcocystis calchasi* seems to be unaffected by acidified drinking water. After its uptake by the intermediate host, infectious sporocysts have to pass through the gastric acid before excysting in the duodenum [40], thus resistance against low pH can be expected. Therefore, it appears unlikely that uptake of sporocysts from contaminated drinking water can be inhibited by addition of acid substances. This correlates with the suggestion of Olias et al. [15] that a transmission via drinking water may be possible. The high prevalence of *S*. *calchasi* in the racing pigeon flocks in Germany (27.8%) suggests a long-established life cycle of *S*. *calchasi* in Germany, probably dating back further than its first discovery in 2006. An ongoing spreading of *S*. *calchasi* in racing pigeon flocks would have resulted in increasing positive flocks towards the end of the present study but was not detected. It seems more likely that cases of PPE in domestic pigeons may have been undetected in former years. During the chronic stage of PPE with neurologic signs, the parasite often is absent in the neural tissue and sarcocysts in the skeletal muscle are the only stage of *S*. *calchasi* that can be detected via histopathology [32]. Since muscular tissue may not have been included in routine examination in cases with a history of neurologic disease, sarcocysts of *S*. *calchasi* may have remained undetected. Also, breeders may have culled birds with neurological signs to prevent spread of a potential APMV-1- or *S*TVC-infection within their flocks without initiating a pathological investigation, leaving the cause of the neurological signs undiscovered.

## Conclusion

In a high proportion of sampled racing pigeon flocks DNA of *S*. *calchasi* was detected, with overall 27.8% of flocks affected, ranging regionally between 0% to 52%. Those flocks were located in 12 out of 13 non-city federal states in Germany. An ongoing spreading was not detected. This suggests a long-lasting parasite-host-relationship between racing pigeons and *Accipiter* hawks in the country. While acidification of drinking water may not be effective to prevent the infection, a fully covered roof is recommended as are other constructional details that help to impede defecation of *Accipiter* hawks into the aviary. It seems essential that testing for *S*. *calchasi* is included in common diagnostic procedures in birds with symptoms possibly associated with PPE.
